# Egg-Laying Behavior of *Cataglyphis niger* Ants Is Influenced More Strongly by Temperature Than Daylength

**DOI:** 10.3390/biology11121714

**Published:** 2022-11-25

**Authors:** Adi Bar, Lior Shalev, Inon Scharf

**Affiliations:** The George S. Wise Faculty of Life Sciences, School of Zoology, Tel Aviv University, Tel Aviv 6997801, Israel

**Keywords:** climate, desert ants, development time, oophagy, oviposition, seasonality

## Abstract

**Simple Summary:**

We examined the contribution of temperature and daylight to egg-laying behavior by queens of desert ants. Decreasing the temperature had a much stronger negative effect on egg laying than shortening the daylight and queens stopped laying eggs. That said, egg laying is plastic, and increasing the temperature swiftly led to its resumption. Next, we showed that moving the colonies to a cooler temperature after eggs already existed, led to egg disappearance, as they were probably eaten by the colony workers. The longer such colonies experience a warmer temperature after eggs are laid, the higher the probability is of such eggs developing into larvae and later into pupae. The workers’ consumption of eggs that fail to develop is evidence of the joint decision of the queen and workers regarding the next generation of workers.

**Abstract:**

Temperature and photoperiod are the two most important factors that affect all aspects of animal life. We conducted two experiments to examine the effect of temperature and photoperiod on egg laying and development in the desert ant *Cataglyphis niger*. In the first experiment, we examined the effect of decreasing temperatures and shortening daylength on egg-laying behavior. An additional treatment was exposure to natural autumn conditions. Decreasing temperatures impaired egg laying much more than shortening daylength. The effect, however, was rapidly reversible when raising the temperature. When the outdoor treatment was brought inside the lab at a suitable temperature, queens started laying eggs as well. In the second experiment, we first kept the colonies under warmer temperatures and moved them gradually to cooler temperatures, 1–20 days after the eggs were laid. The probability of eggs developing into larvae and pupae under cooler temperatures was positively influenced by the exposure duration to warmer temperatures before the temperature switch. When the eggs developed into larvae, longer exposure to warmer temperatures before the temperature switch led to faster development. However, when the eggs disappeared (and were probably eaten), longer exposure to warmer temperatures before the temperature switch led to slower egg disappearance. We suggest that the decision to lay eggs is reversible to some extent because the workers can consume the eggs if conditions deteriorate. We suggest that this reversibility reduces the cost of laying eggs at the wrong time.

## 1. Introduction

Temperature and photoperiod are the most important abiotic factors affecting multiple organismal traits. Decreasing temperatures or shortening days provide a reliable cue for numerous organisms to diapause or migrate to more favorable habitats [1]. Sometimes these two factors interact to affect life-history decisions [2,3]. The environmental direction of change is meaningful on top of the average, i.e., whether conditions slowly improve or deteriorate. For example, plants grow more roots in the ground in which nutrient availability gradually increases than where the resource level is fixed [4]. Temperature and photoperiod are relevant also for social insects. For example, high temperatures lead honeybees to fan their wings and cool the beehive [5]. There is a negative correlation between temperature and diet selectivity in an ant in a temperate region and there is a negative correlation between the usage of recruitment pheromones and temperature [6,7].

Egg laying in social insects is strongly affected by temperature and photoperiod. For example, the Argentine ant (*Linepithema humile* Mayr) lays more eggs with increasing temperatures up to 28 °C, but such eggs survive better at somewhat lower temperatures [8,9]. In bumblebees after winter diapause, more queens laid eggs under long days, and they started doing so earlier [10]. Social insects in seasonal climates often stop laying eggs when summer ends, in a way enabling juveniles either to complete their development before the weather gets cold or to reach the proper stage for diapause [11,12]. It is common to distinguish between ant species, in which winter dormancy is facultative, exogenous, and depends mostly on temperature and photoperiod, and species, in which winter diapause is obligatory, is dictated by endogenous factors, and cannot be prevented by external conditions [13,14].

A good question is how colonies should proceed when conditions unexpectedly deteriorate. Specifically, ant colonies that spend winter without brood but still possess brood when a sudden decrease in temperature occurs can react in two opposite ways: they can either let the brood complete development under suboptimal conditions or allow them to die (and eat them). If completing development is uncertain, it may not be sufficiently beneficial to invest in feeding such larvae. Deteriorating conditions might be correlated with prey shortage, which is a trigger for brood cannibalism in some ants [15,16,17].

Here, we examined the effect of temperature and daylength on egg-laying behavior and the fate of the laid eggs in the desert ant *Cataglyphis niger*. Our study comprised two experiments. The first experiment examined the effect of decreasing temperature and/or shortening daylength and comprised three parts. First, *C. niger* colonies were exposed to either deteriorating temperature or photoperiod. Then, we improved the conditions back to the starting point. We compared the egg-laying behavior of such colonies to other ones kept under natural autumn conditions. Finally, the colonies kept under natural conditions were entered into the lab under the same conditions as the other treatments. We expected egg laying to stop when conditions deteriorate. Photoperiod change could be a more reliable cue for the winter approach [18]. Alternatively, the temperature may play a stronger role as *C. niger* thrives in warm temperatures. We expected the response to take place fast, within days.

The second experiment was based on the results of the first one. Its goal was to examine whether keeping colonies longer at a higher temperature affects the fate of eggs either to disappear or end up as pupae when colonies are moved to a lower temperature. We expected shorter durations at a higher temperature before moving to a lower temperature to increase egg disappearance rate, whereas longer durations should result in larvae and pupae later on.

## 2. Materials and Methods

*Cataglyphis* species are day-active at high temperatures [19,20] and possess several molecular and physiological tools to cope with heat stress, such as a mechanism to maintain cellular integrity [21,22]. They are solitary foragers and lack pheromone-based recruitment while foraging although information on the existence of food nearby triggers workers to leave the nest and forage [23,24]. They use a variety of tools to navigate in their habitat, such as path integration, distance estimation (“odometer”), and panoramic view of the environment, to name only a few [25,26,27]. They easily learn to solve mazes and avoid falling into pitfall traps, pointing to their developed spatial learning skills [24,28,29,30]. The number of larvae in the colony reaches its peak in July/August and decreases in autumn with the shortening day and temperature decrease [31]. In winter, there is no brood in *Cataglyphis* colonies [32]. *Cataglyphis niger* colonies are often polygynous and, in some locations, even super-colonial [33]. Their average colony size in the collection site is ~200 but sizes are quite variable [24,34]. The nest depth can reach one meter or even more, depending on colony size.

**Figure 1:**
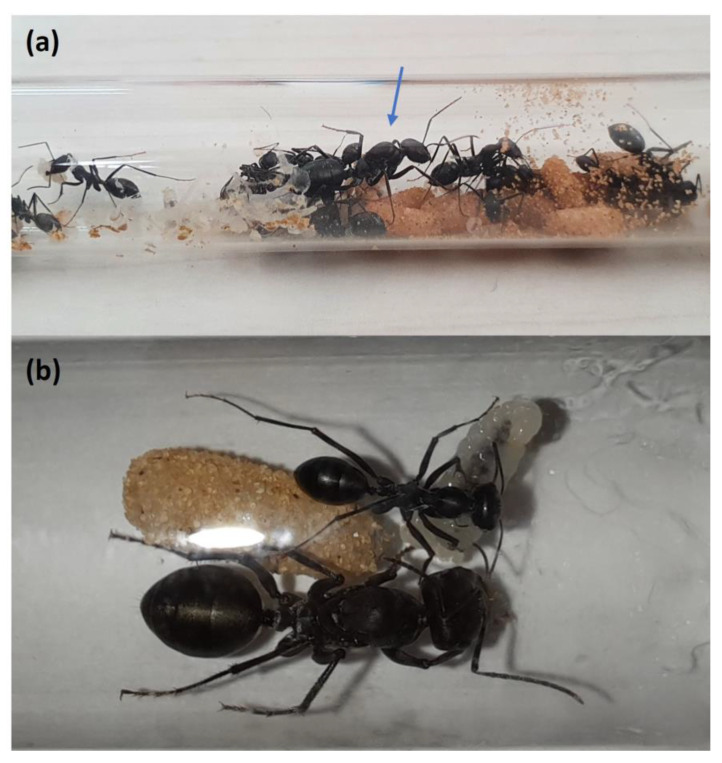
(**a**) A queen (center, marked with an arrow), workers, pupae (right), and a worker holding an egg (left) of the studied species *Cataglyphis niger*. (**b**) A *C. niger* queen (down), worker (up), pupa (left), and larva (right).

We collected 18 and 25 colonies from the Tel Baruch dunes (32.132° N, 34.788° E) for the first and second experiments, respectively. Tel Baruch is a natural sandy area in northwest Tel Aviv (see [35] for its plant and ant species). The average daily maximum and minimum temperatures are 30 °C and 24 °C in July, 27 °C and 20 °C in October, 18 °C and 10 °C in January, and 23 °C and 15 °C in April, respectively (Israel Meteorological Service 2022 https://ims.gov.il/en/ClimateAtlas; accessed in July 2022; measured ~2 km south to Tel Baruch). The colonies were kept for at least five weeks at the laboratory before the experiments commenced. During this period, they were kept on a balcony, exposed to natural late summer conditions and photoperiod. From each colony, we separated the queen plus 20 workers selected randomly (Figure 1). They were moved to a plastic box (26.5 × 16.5 × 11.5 cm; L × W × D, respectively), which contained an aluminum foil shelter, two water tubes, a honey-water mixture (ratio of 3:2) in a single tube, and two *Tenebrio molitor* larvae. The honey-water tube and the larvae were changed every week, and the water tubes were checked twice a week and changed when necessary.

### 2.1. Experiment 1: The Effect of Temperature and Daylength on Oviposition and Development

The experiment comprised three parts, each lasting for three weeks: part A: deteriorating conditions; part B: improving conditions; part C: stable conditions. The colonies were allocated to three different treatments: (1) changing temperature; (2) changing daylength; and (3) outdoor conditions. The colonies in treatments 1 and 2 were kept each in a separate climate cabinet (Rumed, Germany), controlling temperature and daylength. The outdoor treatment was placed outside on a balcony (October–November) during the first two parts (parts A and B) and was brought inside a climate cabinet during part C. The experiment lasted 73 days in total. The outdoor conditions did not fully imitate the natural conditions of nests, as nests are buried in the ground, which can moderate thermal fluctuations at least to some extent.

The initial conditions during part A in the two climate cabinets were 12/12 L:D hours, with 28 °C/23 °C day/night temperature, respectively. In treatment 1, changing temperature, we decreased the temperature twice a week every 3/4 days by 1 °C until the temperature reached 22 °C/17 °C. In treatment 2, changing daylength, we diminished the light period twice a week every 3/4 days by 6-8 min each time until daylight lasted only 11 h 18 m. The outdoor treatment experienced a naturally decreasing temperature due to the ongoing autumn (see the Supplementary Data for temperatures). Regression on the maximal and minimal daily temperatures indicated that in October-November, both temperatures decreased by 0.09 °C–0.14 °C per day. Daylight started at 11 h 32 m (11th October) and ended at 10 h 14 m (29th November). After three weeks, part B commenced, and treatments 1 and 2 increased in temperature and daylength, respectively, at the same rate as the former decrease, until they returned to the conditions at the beginning of part A. After three more weeks, the conditions in treatments 1 and 2 were kept constant, whereas the outdoor treatment was brought into the climate cabinet, in which the temperature formerly changed. At this stage, both climate cabinets maintained the same conditions (28 °C/23 °C and 12:12 L:D; Figure 1b). The experimental aim was to separate the effect of decreasing temperature and shortening daylight. Keeping one factor fixed (either the temperature or the daylight) and changing the other would make the interpretation easier than changing both together. This way, we could isolate the effect of each factor alone.

We noted twice a week whether eggs were laid and removed dead individuals. We later documented the number of pupae and newly emerged workers. We did not count the eggs because they were often laid in batches or attached together, making an exact count impossible. Removing the eggs, separating, and counting them under a stereomicroscope, and bringing them back would have been quite a strong disturbance. The disappearance of eggs after they were noticed is plausibly because workers consumed them. The nests were kept in sealed boxes with no access to the external environment.

Each of the three parts was analyzed separately. We used three χ^2^ tests to compare the presence/absence of eggs among treatments. Throughout the experiment, 12 of the 18 colonies had pupae. We calculated the egg-to-pupa development time by subtracting the first date of egg observation from the first date of pupa observation. We compared the development time between treatments 1 and 2 using a Mann–Whitney test. We omitted treatment 3 because only two colonies produced pupae. Finally, we examined whether worker mortality differed among treatments using a χ^2^ test. The statistical analyses were done using Systat (Systat Software, Inc., San Jose, CA, USA).

### 2.2. Experiment 2: The Effect of Temperature on the Fate of Oviposited Eggs

First, we kept all newly formed colonies at a high temperature of 28 °C/23 °C day/night temperature. Based on experiment 1, such temperatures triggered egg laying. We observed the colonies daily. Second, colonies were moved to a cold temperature of 22 °C/17 °C day/night either 1, 3, 7, 14, or 20 days following the first observation of eggs (2–28 days since the experiment commenced, five colonies in each treatment; Supplementary material). Third, we followed the colonies daily and documented the fate of the eggs. The eggs either disappeared and were probably eaten by workers or progressed into larvae that became pupae later. The experiment lasted two months, and during this timeframe, eight out of ten colonies with larvae ended up with pupae (the two other colonies still contained larvae).

We used logistic regression to examine the effect of the time eggs spent at the high temperature on the fate of eggs (to disappear or to become pupae). Then, we used an ANCOVA to examine whether fate (as a binary variable) interacts with the time eggs spent at the high temperature (a continuous variable) to affect the time until eggs either disappear or turn into larvae. The latter variable was square root transformed because it deviated from a normal distribution.

## 3. Results

### 3.1. Experiment 1: The Effect of Temperature and Daylength on Oviposition and Development

**Figure 2:**
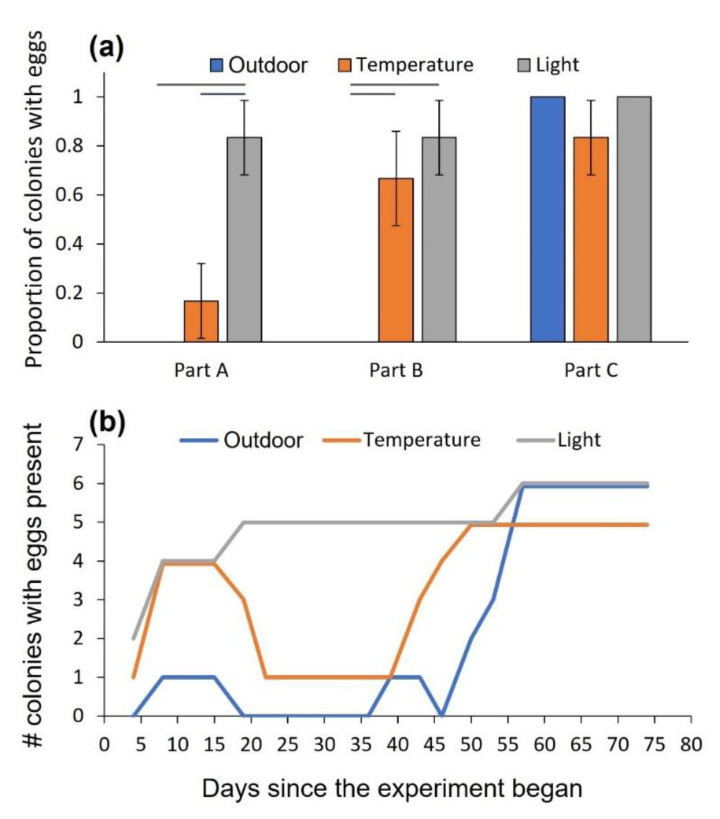
Experiment 1. (**a**) The proportion of colonies (±1 SE) containing eggs at the end of each experimental part. Part A (days 1–24): decreasing temperature and shortening daylight. Part B (days 25–49): increasing temperature and elongating daylight back to the start point. Part C (days 50–74): uniform conditions in all treatments (only day-night fluctuations), bringing the treatment of the outdoor conditions inside the climate cabinet. None of the colonies in the outdoor treatment contained eggs in the first two experimental parts. Gray horizontal lines indicate significant differences between paired treatments. (**b**) The number of colonies producing eggs throughout the experiment as a function of days since its beginning.

When temperature or daylength gradually decreased (part A), more colonies produced eggs in the changing daylength treatment than in the changing temperature and the outdoor treatments (χ^2^ = 10.5, df = 2, *p* = 0.005; Figure 2a). When temperature or daylength gradually increased back to initial conditions (part B), the colonies in the changing temperature treatment caught up and produced as many eggs as the changing daylength treatment, leaving the outdoor treatment behind (χ^2^ = 9.333, df = 2, *p* = 0.009; Figure 2a). After entering the outdoor treatment to the climate cabinet for three weeks (part C), all colonies, except one in which the queen died, had already produced eggs, mitigating the differences among treatments (χ^2^ = 2.118, df = 2, *p* = 0.347; Figure 2a). Queens under cooling conditions laid eggs initially, which were plausibly consumed by the workers as the temperature further declined (Figure 2b).

Egg-to-pupa development time did not differ between the changing temperature and daylength treatments (χ^2^ = 0.900, df = 1, *p* = 0.343; 26.4 ± 7.3 days, mean ± 1 SD). There was also no difference among treatments in the number of dead workers (χ^2^ = 0.541, df = 2, *p* = 0.763; six, five, and three workers died in the changing temperature, changing daylength, and the outdoor treatments, respectively). 

### 3.2. Experiment 2: The Effect of Temperature on the Fate of Oviposited Eggs

**Figure 3:**
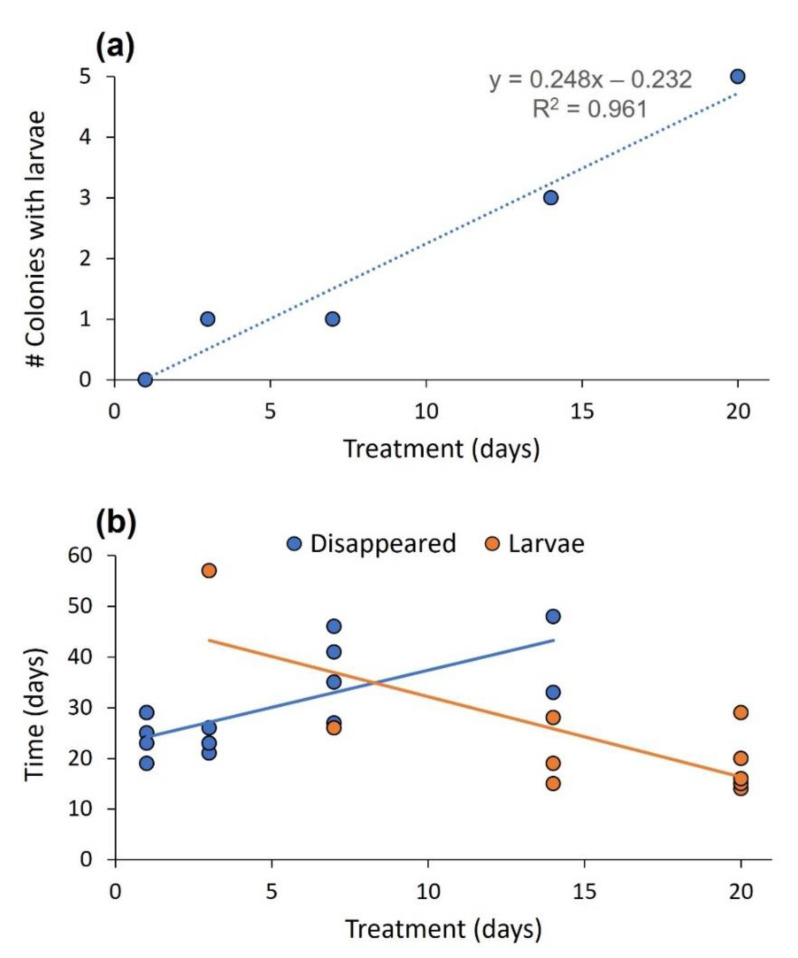
Experiment 2. (**a**) The number of colonies that finished the experiment with larvae (y axis) vs. the treatment, or the number of days spent at the higher temperature after the first appearance of eggs (x axis). The longer the eggs experienced the higher temperature, the higher their probability was to develop into larvae. (**b**) The interactive effect of the treatment and the egg fate, i.e., to become larvae or disappear, on the time elapsed until reaching one of the two situations.

The time eggs spent at the high temperature had a strong effect on the fate of eggs: shorter durations of the high temperature before moving to the cold temperature led to the disappearance of eggs, whereas longer durations led to eggs turning into larvae (Z = −2.854, *p* = 0.004; Figure 3a). Moreover, the time eggs spent at the high temperature interacted with fate to affect the time until eggs either disappeared or turned into pupae (fate × treatment interaction: F_1,21_ = 23.762, *p* < 0.001; Figure 3b). If eggs ended up as larvae, longer durations at a higher temperature shortened the time required to turn into larvae after moving to the low temperature. In contrast, if eggs disappeared, longer durations at a high temperature elongated the time until disappearance. To complete the analysis, fate as the main effect was significant (longer time required to turn into larvae; F_1,21_ = 10.318, *p* = 0.004) but treatment was not (F_1,21_ = 0.044, *p* = 0.835).

## 4. Discussion

Our first experiment demonstrated that decreasing temperatures prevented egg laying in *C. niger*, whereas shortening daylength did not. When the temperature was elevated back to the initial level, colonies started laying eggs as well. Finally, colonies that moved from natural conditions to a high temperature laid eggs too. All the changes took place within days. The second experiment showed that eggs in colonies moved to a cooler temperature either turn into larvae (and later pupate) or disappear and are probably eaten by workers. Their fate depended strongly on the time elapsed since eggs were laid and the temperature switch: the longer the colonies were exposed to higher temperatures, the higher the probability was that the eggs would end up as larvae and later as pupae. Finally, it took longer for eggs to disappear at the lower temperature the longer they were exposed to the higher temperature before the temperature switch.

Temperature plays a more important role than photoperiod in *C. niger*, at least under lab conditions, as previously suggested and demonstrated in ant species of temperate regions [13,36] as well as other insects, such as chrysomeloid beetles [3]. There are several possible explanations. First, photoperiod may be more important in temperate regions than in Mediterranean ones, because the seasonal changes in photoperiod are greater there [37]. That said, the habitat-of-origin in the current experiment still experiences significant seasonal fluctuations in daylength (14 and 10 h of daylight hours in summer and winter, respectively), which can serve as a reliable cue. Second, *Cataglyphis* spp. are heat adapted and inactive in winter [20,38]. Third, the decreasing temperature might pose a problem for egg production per se on top of being a negative cue for approaching winter [39]. We suggest that the third explanation is less likely here because the seasonal differences in the nest below ground level are plausibly much more moderate than on the ground.

The response to both improving and deteriorating conditions was fast, i.e., in a matter of days. This result suggests that *Cataglyphis niger* does not require a cold period before ovarian reactivation and that it is easily affected by exogenous factors like temperature. The observed pattern contrasts with ant species from more temperate regions, such as the high-mountain ant *Proformica longiseta*, which requires undergoing a cold period [40]. Fast response to increasing temperatures in spring may be adaptive, because *Cataglyphis* spp. prey on dead arthropods [19], which are more abundant in spring than summer [41].

As our data indicate the egg-to-pupa development time is about a month, it could be profitable to start reproducing as early as possible in order not to miss the season’s peak. Moreover, as the data indicate, “mistakes” in egg laying are easily reversible, as the workers may consume the eggs if they deem the conditions unsuitable for larval development or if the eggs fail to develop into larvae. Thus, it probably makes sense to lay eggs, and let them develop if conditions stay warm. Alternatively, the workers can terminate the eggs’ development if conditions deteriorate although egg laying incurs some cost to the queen (as suggested also by [36]). The mechanism could be the time it requires for the eggs to hatch: we suggest that when they do not hatch after some time threshold, they are consumed by the workers. The decision of whether to lay eggs at the end of summer can be analyzed using the common trade-off between type I and type II errors in animal behavior, such as that regarding the mating partner or nestmate recognition [42,43]. If the cost of a false positive decision (laying eggs although the time is unsuitable) is lower than that of a false negative one (not laying eggs although the time for development is sufficient), then queens should lay eggs quite late in the season. The ability to consume the eggs moderates the cost of such a false positive decision. A similar mechanism of egg eating takes place under starvation when eggs and larvae are either consumed by the workers or fed to other larvae [17,44]. Egg cannibalism is common in social insects and may be unrelated to colony starvation, with one reason being relatedness considerations. For example, queens in polygynous colonies eat eggs (hopefully of rival queens and not their own), or workers eat eggs laid by other workers or feed them to larvae [45,46,47]. Another reason could be the use of eggs as an especially suitable food source for small larvae [16]. In some other ant species, larvae are also cannibalized and serve as a nutrient source (“larvae as storage vessels”; [48,49]).

A good question is how workers should “know” whether to eat the eggs or let them develop. The second experiment suggests there is a correlation between the time eggs spend at a warm temperature and the giving-up time of workers after temperatures cool down. Alternatively, it could also be that the eggs are eaten only if they are perceived as not viable, and eggs remain viable longer under cooler temperatures if they first go through a longer time at warmer temperatures. These are all suggestions that remain to be tested. The thermal and photoperiod changes applied here were gradual. It is interesting to examine what should happen when the change is abrupt and how fast such changes should last to have an impact. In addition, a combination of further temperatures and photoperiods may lead to an interactive effect of the two. For example, whereas temperature is more influential on egg laying under the conditions tested, the temperature might be less important when daylight is shorter. We hope our experiment sheds light on the conditions required to successfully raise *Cataglyphis* colonies under laboratory conditions and on the strong thermal effect on oviposition and larval development.

## 5. Conclusions

Our study indicates that egg laying in *C. niger* ants is much more strongly affected by reducing or increasing the temperature than daylength. The change in egg-laying behavior is fast, within a few days. The longer eggs experience a warmer temperature, the higher their chances are to develop into larvae. Furthermore, if eggs disappear (probably eaten by workers), the time until such disappearance is shorter if the eggs were kept for a shorter period at a warmer temperature. These results demonstrate that egg-laying behavior and the eggs’ fate are plastic and easily reversible, potentially enabling the colonies to adjust rapidly to seasonal changes.

## Data Availability

The dataset is attached as an Excel file (see Supplementary Material).

## Supplementary Materials

The following supporting information can be downloaded at: https://www.mdpi.com/article/10.3390/biology11121714/s1, Figure S1: Daily maximum and minimum temperatures during Experiment 1. The dataset is also available.

## References

[1] Dingle H. (1972). Migration strategies of insects: Migration is an environmentally modified physiological syndrome adapted for dispersal and colonization. Science.

[2] Saunders D.S. (1971). The temperature-compensated photoperiodic clock ‘programming’ development and pupal diapause in the flesh-fly, *Sarcophaga argyrostoma*. J. Insect Physiol..

[3] Kutcherov D.A., Lopatina E.B. (2022). Photoperiodic insensitivity of temperature-dependent development in some chrysomeloid beetles (Coleoptera: Chrysomelidae, Megalopodidae). Entomol. Rev..

[4] Shemesh H., Arbiv A., Gersani M., Ovadia O., Novoplansky A. (2010). The effects of nutrient dynamics on root patch choice. PLoS ONE.

[5] Southwick E.E., Heldmaier G. (1987). Temperature control in honey bee colonies. Bioscience.

[6] Traniello J.F., Fujita M.S., Bowen R.V. (1984). Ant foraging behavior: Ambient temperature influences prey selection. Behav. Ecol. Sociobiol..

[7] Ruano F., Tinaut A., Soler A.J.J. (2000). High surface temperatures select for individual foraging in ants. Behav. Ecol..

[8] Abril S., Oliveras J., Gómez C. (2008). Effect of temperature on the oviposition rate of Argentine ant queens (*Linepithema humile* Mayr) under monogynous and polygynous experimental conditions. J. Insect Physiol..

[9] Abril S., Oliveras J., Gómez C. (2010). Effect of temperature on the development and survival of the Argentine ant, *Linepithema humile*. J. Insect Sci..

[10] Tasei J.N., Aupinel P. (1994). Effect of photoperiodic regimes on the oviposition of artificially overwintered *Bombus terrestris* L. queens and the production of sexuals. J. Apic. Res..

[11] Kipyatkov V.E., Lopatina E.B. (1997). Seasonal cycle and winter diapause induction in ants of the genus Myrmica in the Polar Circle region. Proc. Int. Colloq. Soc. Insects.

[12] Foitzik S., Heinze J. (2000). Intraspecific parasitism and split sex ratios in a monogynous and monandrous ant (*Leptothorax nylanderi*). Behav. Ecol. Sociobiol..

[13] Kipyatkov V.E. (2001). Seasonal life cycles and the forms of dormancy in ants (Hymenoptera: Formicoidea). Acta Soc. Zool. Bohem..

[14] Lopatina E., Shields V.D.C. (2018). Structure, diversity and adaptive traits of seasonal cycles and strategies in ants (ch. 2). The Complex World of Ants.

[15] Sorensen A.A., Busch T.M., Vinson S.B. (1983). Factors affecting brood cannibalism in laboratory colonies of the imported fire ant, *Solenopsis invicta* Buren (Hymenoptera: Formicidae). J. Kansas Entomol. Soc..

[16] Masuko K. (2003). Larval oophagy in the ant *Amblyopone silvestrii* (Hymenoptera, Formicidae). Insectes Sociaux.

[17] Rüppell O., Kirkman R.W. (2005). Extraordinary starvation resistance in *Temnothorax rugatulus* (Hymenoptera, Formicidae) colonies: Demography and adaptive behavior. Insectes Sociaux.

[18] Hesse L., Falk J., Koch K. (2016). Inflexible versus flexible: The influence of temperature and photoperiod on pre- and post-eyespot development time in Libellulidae (Odonata). Physiol. Entomol..

[19] Lenoir A., Aron S., Cerdá X., Hefetz A. (2009). *Cataglyphis* desert ants: A good model for evolutionary biology in Darwin’s s anniversary year: A review. Isr. J. Entomol..

[20] Boulay R., Aron S., Cerdá X., Doums C., Graham P., Hefetz A., Monnin T. (2017). Social life in arid environments: The case study of *Cataglyphis* ants. Annu. Rev. Entomol..

[21] Gehring W.J., Wehner R. (1995). Heat shock protein synthesis and thermotolerance in *Cataglyphis*, an ant from the Sahara desert. Proc. Nat. Acad. Sci. USA.

[22] Perez R., de Souza Araujo N., Defrance M., Aron S. (2021). Molecular adaptations to heat stress in the thermophilic ant genus *Cataglyphis*. Mol. Ecol..

[23] Razin N., Eckmann J.P., Feinerman O. (2013). Desert ants achieve reliable recruitment across noisy interactions. J. R. Soc. Interface.

[24] Bega D., Samocha Y., Yitzhak N., Saar M., Subach A., Scharf I. (2020). Non-spatial information on the presence of food elevates search intensity in ant workers, leading to faster maze solving in a process parallel to spatial learning. PLoS ONE.

[25] Müller M., Wehner R. (1994). The hidden spiral: Systematic search and path integration in desert ants, *Cataglyphis fortis*. J. Comp. Physiol. A.

[26] Thiélin-Bescond M., Beugnon G. (2005). Vision-independent odometry in the ant *Cataglyphis cursor*. Naturwissenschaften.

[27] Wehner R., Müller M. (2006). The significance of direct sunlight and polarized skylight in the ant’s celestial system of navigation. Proc. Nat. Acad. Sci. USA.

[28] Chameron S., Schatz B., Pastergue-Ruiz I., Beugnon G., Collett T.S. (1998). The learning of a sequence of visual patterns by the ant *Cataglyphis cursor*. Proc. R. Soc. B.

[29] Wystrach A., Buehlmann C., Schwarz S., Cheng K., Graham P. (2020). Rapid aversive and memory trace learning during route navigation in desert ants. Curr. Biol..

[30] Bar A., Marom C., Zorin N., Gilad T., Subach A., Foitzik S., Scharf I. (2022). Desert ants learn to avoid pitfall traps while foraging. Biology.

[31] Cerdá X., Retana J., De Haro A. (1994). Social carrying between nests in polycalic colonies of the monogynous ant *Cataglyphis iberica* (Hymenoptera: Formicidae). Sociobiology.

[32] Darras H., Kuhn A., Aron S. (2014). Genetic determination of female castes in a hybridogenetic desert ant. J. Evol. Biol..

[33] Brodetzki T.R., Inbar S., Cohen P., Aron S., Privman E., Hefetz A. (2019). The Interplay between Incipient species and social polymorphism in the Desert Ant *Cataglyphis*. Sci. Rep..

[34] Subach A., Avidov B., Dorfman A., Bega D., Gilad T., Kvetny M., Reshef M.H., Foitzik S., Scharf I. (2023). The value of spatial experience and group size for ant colonies in direct competition. Insect Sci..

[35] Saar M., Subach A., Reato I., Liber T., Pruitt J.N., Scharf I. (2018). Consistent differences in foraging behavior in 2 sympatric harvester ant species may facilitate coexistence. Curr. Zool..

[36] Nakamura K., Fujiyama M., Ohta K. (2017). Effect of temperature on queen oviposition and seasonal colony development in *Lasius japonicus* (Hymenoptera: Formicidae). Appl. Entomol. Zool..

[37] Bradshaw W.E., Holzapfel C.M. (2007). Evolution of animal photoperiodism. Annu. Rev. Ecol. Evol. Syst..

[38] Wehner R., Wehner S. (2011). Parallel evolution of thermophilia: Daily and seasonal foraging patterns of heat-adapted desert ants: *Cataglyphis* and *Ocymyrmex* species. Physiol. Entomol..

[39] Berger D., Walters R., Gotthard K. (2008). What limits insect fecundity? Body size-and temperature-dependent egg maturation and oviposition in a butterfly. Funct. Ecol..

[40] Fernández-Escudero I.F., Tinaut A., Ruano F. (1997). Ovarian maturation under cold winter conditions in a high-mountain ant (Hymenoptera: Formicidae). Environ. Entomol..

[41] Doblas-Miranda E., Sánchez-Piñero F., González-Megías A. (2007). Soil macroinvertebrate fauna of a Mediterranean arid system: Composition and temporal changes in the assemblage. Soil Biol. Biochem..

[42] Scharf I., Martin O.Y. (2013). Same-sex sexual behavior in insects and arachnids: Prevalence, causes, and consequences. Behav. Ecol. Sociobiol..

[43] Rossi N., Baracchi D., Giurfa M., d’Ettorre P. (2019). Pheromone-induced accuracy of nestmate recognition in carpenter ants: Simultaneous decrease in type I and type II errors. Am. Nat..

[44] Modlmeier A.P., Foitzik S., Scharf I. (2013). Starvation endurance in the ant *Temnothorax* nylanderi depends on group size, body size and access to larvae. Physiol. Entomol..

[45] Bourke A.F. (1991). Queen behaviour, reproduction and egg cannibalism in multiple-queen colonies of the ant *Leptothorax acervorum*. Anim. Behav..

[46] Heinze J., Trunzer B., Oliveira P.S., Hölldobler B. (1996). Regulation of reproduction in the neotropical ponerine ant, *Pachycondyla villosa*. J. Insect Behav..

[47] Monnin T., Peeters C. (1997). Cannibalism of subordinates’ eggs in the monogynous queenless ant *Dinoponera quadriceps*. Naturwissenschaften.

[48] Nonacs P. (1991). Less growth with more food: How insect-prey availability changes colony demographics in the ant, *Camponotus floridanus*. J. Insect Physiol..

[49] Kaspari M., Vargo E.L. (1995). Colony size as a buffer against seasonality: Bergmann’s rule in social insects. Am. Nat..

